# Performance Analysis of Non-Orthogonal Multiple Access-Enhanced Autonomous Aerial Vehicle-Assisted Internet of Vehicles over Rician Fading Channels

**DOI:** 10.3390/e27090907

**Published:** 2025-08-27

**Authors:** Zheming Zhang, Yixin He, Yifan Lei, Zehui Cai, Fanghui Huang, Xingchen Zhao, Dawei Wang, Lujuan Li

**Affiliations:** 1 College of Information Science and Engineering, Jiaxing University, Jiaxing 314001, China; 00199895@stu.zjxu.edu.cn (Z.Z.); 00210713@stu.zjxu.edu.cn (Y.L.); 2 Sino-European Joint Lab for Health Information Processing and Applications, Jiaxing University, Jiaxing 314001, China; 3 College of Artificial Intelligence, Jiaxing University, Jiaxing 314001, China; 00193437@zjxu.edu.cn (Z.C.); huangfanghui@zjxu.edu.cn (F.H.); 4 College of Mechanical Engineering, Jiaxing University, Jiaxing 314001, China; wusaqi@zjxu.edu.cn; 5 School of Electronics and Information, Northwestern Polytechnical University, Xi’an 710072, China; wangdw@nwpu.edu.cn

**Keywords:** autonomous aerial vehicles (AAVs), full-duplex non-orthogonal multiple access (FD-NOMA), Internet of Vehicles (IoV), Rician fading, vehicle-to-everything (V2X)

## Abstract

The increasing number of intelligent connected vehicles (ICVs) is leading to a growing scarcity of spectrum resources for the Internet of Vehicles (IoV), which has created an urgent need for the use of full-duplex non-orthogonal multiple access (FD-NOMA) techniques in vehicle-to-everything (V2X) communications. Meanwhile, for the flexibility of autonomous aerial vehicles (AAVs), V2X communications assisted by AAVs are regarded as a potential solution to achieve reliable communication between ICVs. However, if the integration of FD-NOMA and AAVs can satisfy the requirements of V2X communications, then quickly and accurately analyzing the total achievable rate becomes a challenge. Motivated by the above, an accurate analytical expression for the total achievable rate over Rician fading channels is proposed to evaluate the transmission performance of NOMA-enhanced AAV-assisted IoV with imperfect channel state information (CSI). Then, we derive an approximate expression with the truncated error, based on which the closed-form expression for the approximate error is theoretically provided. Finally, the simulation results demonstrate the accuracy of the obtained approximate results, where the maximum approximate error does not exceed 0.5%. Moreover, the use of the FD-NOMA technique in AAV-assisted IoV can significantly improve the total achievable rate compared to existing work. Furthermore, the influence of key network parameters (e.g., the speed and Rician factor) on achievable rate is thoroughly discussed.

## 1. Introduction

As a core component of intelligent transportation systems (ITSs), Internet of Vehicles (IoV) can sense the traffic environment in real time, optimize route planning, improve driving safety and reduce energy consumption through vehicle-to-everything (V2X) techniques [1,2,3]. However, the current IoV architecture is limited by the static deployment characteristics of ground base stations (BSs), resulting in coverage holes in urban canyons and remote areas [4,5,6]. In addition, spectrum resource competition in high-density vehicular environments can easily lead to a deterioration in the quality of service (QoS). Facing these challenges, the introduction of autonomous aerial vehicles (AAVs) to V2X techniques offers a potential solution for IoV [7,8,9]. Leveraging the three-dimensional mobility of AAVs, dynamic air-to-ground collaborative networks can be rapidly established to avoid signal blind spots caused by terrain occlusion or emergency events.

In AAV-assisted IoV, the implementation of full-duplex non-orthogonal multiple access (FD-NOMA) effectively breaks through the resource utilization bottlenecks of conventional orthogonal multiple access (OMA) schemes [10,11,12]. FD-NOMA techniques support non-orthogonal superimposed transmission of multiple intelligent connected vehicle (ICVs) in the same time-frequency resource block through joint multiplexing of power domain and code domain [13,14,15,16]. Concurrently, the full-duplex technique enables AAVs to simultaneously transmit and receive signals, eliminating the timeslot switching overhead associated with traditional half-duplex operations and reducing end-to-end communication latency. Therefore, NOMA-enhanced AAV-assisted IoV provides an innovative technical pathway to satisfy demanding requirements of 6G-enabled IoV evolution, including massive connectivity, ultra-high reliability, and deterministic latency.

Several recent studies have been conducted on AAV-assisted vehicular communications and FD-NOMA-enabled V2X techniques. For instance, the authors in [17] presented a FD-NOMA-based V2X communication mechanism, and focused on its capacity performance analysis. Inspired by this work, an average sum rate maximization scheme for AAV-assisted networks was proposed, which jointly considered resource management and trajectory optimization [18]. Moreover, the authors in [19,20,21,22] investigated the application of NOMA techniques and AAVs in IoV, aiming to enhance the autonomous driving performance of vehicle platoons. By adopting network slicing, the authors in [23] proposed a resource allocation scheme for AAV-assisted V2X communications, where multiple flexible AAVs were deployed as aerial base stations (BSs) to assist ground BSs in providing vehicle-related services to ICVs. Furthermore, some efforts have been devoted to the relay-aided V2X communication [24,25], downlink and uplink cooperative transmission [26,27], connectivity analysis [28,29], and multi-dimensional resource allocation [30,31].

The above studies have facilitated the integration of AAVs and NOMA into the IoV, thereby supporting high-capacity, ultra-reliable, and low-latency vehicular information exchange. However, several challenges remain to be addressed. First, the authors make an implicit assumption in [17,18,19,20,23] that the channel state information (CSI) can be accurately obtained during performance analysis or resource allocation, ignoring the mobility of AAVs and ICVs. Second, although the authors in [25,27,28,30,31] consider the effect of imperfect CSI on AAV-assisted IoV, they use empirical formulas directly to describe the channel gain for the purpose of simplifying the model. By contrast, the authors in [24] investigate the impact of Rayleigh fading on NOMA-enhanced AAV-assisted IoV, but Rician fading is more appropriate for characterizing air-to-ground vehicular communication due to its strong line-of-sight (LoS) path. Finally, analyzing the transmission performance of NOMA-enhanced AAV-assisted IoV is a challenge due to the complex exponential integral functions associated with Rician fading channels.

Motivated by the above, we employ the FD-NOMA technique within AAV-assisted IoV to satisfy the demands of large-scale on-board connected devices and differentiated QoS in V2X communications. First, by taking the imperfect CSI and Rayleigh fading into account, we focus on the performance analysis of V2X downlinks, with the goal of addressing a key issue: how to quickly and accurately analyze the total achievable rate of V2X communications in NOMA-enhanced AAV-assisted IoV. To solve this problem, we propose an accurate analytical expression of the total achievable rate over Rician fading channels, where the least squares (LS) method is employed to estimate CSI. Then, since the proposed expression involves improper integrals and summations of infinite series, we derive an approximate expression to simplify the calculation. We theoretically analyze that the approximate error is determined by the truncated series. Finally, the simulation results show that the FD-NOMA technique outperforms the FD-OMA technique in terms of total achievable rate in AAV-assisted V2X communications. Additionally, by using Monte Carlo experiments, we find that the derived approximate expression closely matches the proposed accurate analytical expression, with the maximum approximate error not exceeding 0.5%.

## 2. V2X Communication Model

**Figure 1:**
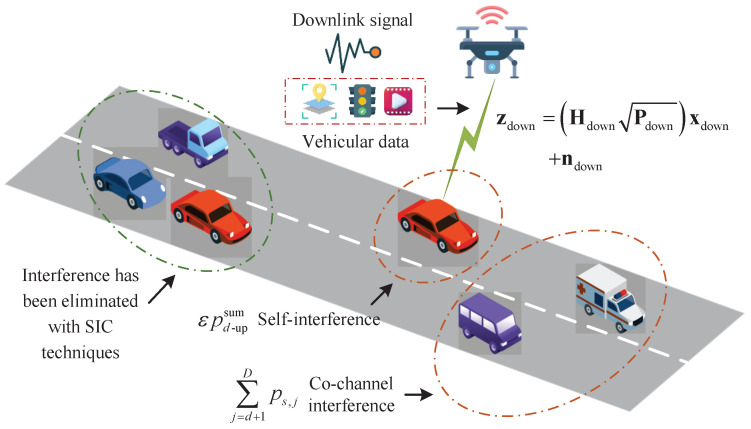
NOMA-enhanced AAV-assisted IoV.

Figure 1 illustrates the V2X communication model considered in the NOMA-enhanced AAV-assisted IoV scenario, which consists of *U* AAVs and *V* ICVs. The sets of AAVs and ICVs are defined as $U=\left\{1,\ldots ,U\right\}$ and $V=\left\{1,\;\ldots ,\;V\right\}$, respectively. Additionally, the FD-NOMA technique is employed in AAV-assisted IoV to improve the transmission performance of downlink [10]. In NOMA-enhanced AAV-assisted IoV, we select *S* source nodes (SNs), defined as $S=\left\{1,\;\ldots ,\;S\right\}$, and *D* destination nodes (DNs), defined as $D=\left\{1,\ldots ,D\right\}$, for performance analysis. It should be noted that the SN and DN can be AAVs or ICVs. The matrix $H_{\mathrm{down}}$ is defined as the downlink channel matrix between *S* SNs and *D* DNs, which can be expressed as

(1) $$\begin{array}{c} H_{\mathrm{down}}={\left[h_{1}^{\mathrm{down}},\ldots ,h_{d}^{\mathrm{down}},\ldots ,h_{D}^{\mathrm{down}}\right]}^{T}, \end{array}$$

where $H_{\mathrm{down}}\in C^{S\times D}$, and ${\left[\cdot \right]}^{T}$ is the transposed matrix of the matrix $\left[\cdot \right]$. In (1), $h_{d}^{\mathrm{down}}$ is the channel matrix from *S* SNs to the *d*-th $\left(\forall d\in D\right)$ SN, $h_{d}^{\mathrm{down}}=\left[h_{1,d}^{\mathrm{down}},\ldots ,h_{s,d}^{\mathrm{down}},\ldots ,h_{S,d}^{\mathrm{down}}\right]$, where $h_{s,d}^{\mathrm{down}}$ is the downlink channel fading coefficient from the *s*-th $\left(\forall s\in S\right)$ SN to the *d*-th DN. Therefore, the downlink received signal $z_{\mathrm{down}}$ is given by

(2) $$\begin{array}{c} z_{\mathrm{down}}=\left(H_{\mathrm{down}}\sqrt{P_{\mathrm{down}}}\right)x_{\mathrm{down}}+n_{\mathrm{down}}, \end{array}$$

where $\sqrt{P_{\mathrm{down}}}$ is the downlink power matrix, $x_{\mathrm{down}}$ is the downlink transmission signal matrix, and $n_{\mathrm{down}}$ is the downlink noise matrix. We have $\sqrt{P_{\mathrm{down}}},\;x_{\mathrm{down}}C\in^{D\times 1}$ and $n_{\mathrm{down}}∼CN\left(0,\;\sigma_{\mathrm{down}}^{2}I_{D}\right)$, where $\sigma_{\mathrm{down}}^{2}$ is the downlink noise power and $I_{D}$ is the identity matrix. Based on the time-reversal symmetry of channels, we assume that the uplink and downlink channel matrices can be transposed of each other [27]. The matrix $H_{\mathrm{up}}$ is defined as the uplink channel matrix, and we can get $H_{\mathrm{up}}={\left(H_{\mathrm{down}}\right)}^{T}$. Similarly, the uplink received signal $z_{\mathrm{up}}$ is given by

(3) $$\begin{array}{c} z_{\mathrm{up}}=\left(H_{\mathrm{up}}\sqrt{P_{\mathrm{up}}}\right)x_{\mathrm{up}}+n_{\mathrm{up}}, \end{array}$$

where $x_{\mathrm{up}}$ is the uplink transmission signal matrix, $P_{\mathrm{up}}={\left(P_{\mathrm{down}}\right)}^{T}$, and $n_{\mathrm{up}}$ is the uplink noise matrix, where $n_{\mathrm{up}}∼CN\left(0,\;\sigma_{\mathrm{up}}^{2}I_{S}\right)$ and $\sigma_{\mathrm{up}}^{2}$ is the uplink noise power. Thus, the total power received by the *d*-th DN can be calculated as $p_{d-\mathrm{down}}^{\mathrm{sum}}=\sum_{s=1}^{S}p_{s,d}^{\mathrm{down}}$. The self-interference power $p_{d-\mathrm{up}}^{\mathrm{sum}}$ can be calculated as $p_{d-\mathrm{up}}^{\mathrm{sum}}=\sum_{d=1}^{D}p_{s,d}^{\mathrm{up}}$.

As mentioned above, the total achievable rate $R_{\mathrm{tot}}$ is

(4) $$\begin{array}{c} R_{\mathrm{tot}}=\sum_{s=1}^{S}\sum_{d=1}^{D}\log_{2}\left(1+\frac{p_{s,d}^{\mathrm{down}}{\left|h_{s,d}\right|}^{2}}{\varepsilon p_{d-\mathrm{up}}^{\mathrm{sum}}+\sum_{j=d+1}^{D}p_{s,j}+\sigma^{2}}\right), \end{array}$$

where $\sum_{j=d+1}^{D}p_{s,j}$ is the co-channel interference, $\varepsilon \in \left[0,\;1\right]$ is the self-interference coefficient, and $\sigma^{2}$ is the noise power. In addition, in NOMA-enhanced AAV-assisted IoV, it is assumed that the channel gain is monotonically increasing, i.e., ${\left|h_{s,1}\right|}^{2}\leq {\left|h_{s,2}\right|}^{2},\ldots ,{\left|h_{s,\;D}\right|}^{2}$. In this situation, after the *d*-th DN performs the successive interference cancellation (SIC) technique, its co-channel interference comes from the $\left(s+1\right)$-th SN to the *S*-th SN [30].

Furthermore, due to the mobility of AAVs and ICVs, the DN is unable to accurately and real-time acquire the perfect CSI [32]. To address this, we adopt the LS method to estimate CSI over Rician fading channels. Taking $h_{s,d}^{\mathrm{down}}$ as an example, the estimation process is as follows.

(5) $$\begin{array}{c} h_{s,d}^{\mathrm{down}}\left(t\right)=\sqrt{\frac{K}{K+1}}{\bar{h}}_{s,d}^{\mathrm{down}}\left(t\right)+\sqrt{\frac{1}{K+1}}{\tilde{h}}_{s,d}^{\mathrm{down}}\left(t\right), \end{array}$$

where *K* is the Rician factor, ${\bar{h}}_{s,d}^{\mathrm{down}}\left(t\right)$ is the LoS component, and ${\tilde{h}}_{s,d}^{\mathrm{down}}\left(t\right)$ is the non-line-of-sight (NLoS) component. Specifically, ${\bar{h}}_{s,d}^{\mathrm{down}}\left(t\right)$ is affected by the speed of AAVs and ICVs, which can be further expressed as ${\bar{h}}_{s,d}^{\mathrm{down}}\left(t\right)=e^{\left(2\pi f_{d}t+\varphi_{0}\right)k}$, where $f_{d}$ is the Doppler shift, and $\varphi_{0}$ is the initial phase. In addition, ${\tilde{h}}_{s,d}^{\mathrm{down}}\left(t\right)$ is the superposition of *N* scattering paths, which can be further expressed as ${\tilde{h}}_{s,d}^{\mathrm{down}}\left(t\right)=\sum_{n=1}^{N}\alpha_{n}e^{\left(2\pi f_{d}^{n}t+\varphi_{n}\right)k}$, where $\alpha_{n}$, $f_{d}^{n}$, and $\varphi_{n}$ are the amplitude, Doppler shift, and initial phase of the *n*-th scattering path, respectively. Moreover, the Doppler shift $f_{d}$ can be calculated as $f_{d}=\frac{v_{1}+v_{2}}{\lambda }\cos\left(\theta \right)$, where λ is the wavelength, θ is the angle of arrival, and $v_{1}$ and $v_{2}$ are the speeds of AAVs and ICVs, respectively. By using the LS method, the estimation problem can be formulated as

(6) $${\overset{⏜}{h}}_{s,d}^{\text{down}}(t)=\underset{h_{s,d}^{\text{down}}(t)}{\arg\min}\sum_{m=1}^{M}{\left|z\left(t_{n}\right)-h_{s,d}^{\text{down}}\left(t_{n}\right)x\left(t_{n}\right)\right|}^{2},$$

where *M* is the sampling points. Then, this problem is transformed into the matrix form. Based on this, the channel gain estimation is calculated by using the LS method, where the Doppler shift is updated according to $v_{1}$ and $v_{2}$.

The notations utilized in this paper are summarized in Table 1.

## 3. Total Achievable Rate Analysis

### 3.1. Accurate Analytical Expression

To further analyze the achievable rate in the NOMA-enhanced AAV-assisted IoV scenario, we rewrite the Rician factor *K* to determine the proportional relationship between LoS and NLoS components. Specifically, we have $K=\frac{{\left(\kappa_{\mathrm{LoS}}\right)}^{2}}{2{\left(\kappa_{\mathrm{NLoS}}\right)}^{2}}$, where ${\left(\kappa_{\mathrm{LoS}}\right)}^{2}$ is the channel gain of the LoS component, and $2{\left(\kappa_{\mathrm{NLoS}}\right)}^{2}$ is the average channel gain of all NLoS components. Additionally, let $\gamma_{s,d}$ denote the instantaneous signal-to-interference-plus-noise ratio (SINR). As shown in (4), the average SINR $E\left(\gamma_{s,d}\right)$ of each DN can be expressed as

(7) $$\begin{array}{c} E\left(\gamma_{s,d}\right)=\frac{p_{s,d}^{\mathrm{down}}}{\varepsilon p_{d-\mathrm{up}}^{\mathrm{sum}}+\sum_{j=d+1}^{D}p_{s,j}+\sigma^{2}}. \end{array}$$

As discussed in [10], the probability density function $f\left(\gamma_{s,d}\right)$ of $E\left(\gamma_{s,d}\right)$ is

(8) $$\begin{array}{ccc} f\left(\gamma_{s,d}\right)\; & = & \;\exp\left\{\frac{-{\left(\kappa_{\mathrm{LoS}}\right)}^{2}}{2{\left(\kappa_{\mathrm{NLoS}}\right)}^{2}}-\gamma_{s,d}\varpi \left[E\left(\gamma_{s,d}\right)\right]\right\}\\ \; & & \;\times \varpi \left[E\left(\gamma_{s,d}\right)\right]I_{0}\left[E\left(\gamma_{s,d}\right)\right], \end{array}$$

where $I_{0}\left[\cdot \right]$ is the first-kind zero-order Bessel function, and we have

(9) $$\begin{array}{c} \varpi \left[E\left(\gamma_{s,d}\right)\right]=\frac{{\left(\kappa_{\mathrm{LoS}}\right)}^{2}+2{\left(\kappa_{\mathrm{NLoS}}\right)}^{2}}{2{\left(\kappa_{\mathrm{NLoS}}\right)}^{2}E\left(\gamma_{s,d}\right)}, \end{array}$$

and

(10) $$\begin{array}{c} I_{0}\left[E\left(\gamma_{s,d}\right)\right]=I_{0}\left[2\sqrt{\frac{\varpi \left[E\left(\gamma_{s,d}\right)\right]\gamma_{s,d}}{2{\left(\kappa_{\mathrm{NLoS}}\right)}^{2}{\left(\kappa_{\mathrm{LoS}}\right)}^{-2}}}\right]. \end{array}$$

Note that (7) calculates the average SINR over the fading channel realizations. On the other hand, (8) calculates the distribution of SINR at a given instant. It gives us the probability of observing a particular SINR value at a specific time or under a certain realization of the fading gains.

The achievable rate $R_{s,d}$ from the *s*-th SN to the *d*-th DN is given by

(11) $$\begin{array}{ccc} R_{s,d}\; & = & \;E\left\{\log_{2}\left[1+E\left(\gamma_{s,d}\right)\right]\right\}\\ \; & = & \;\int_{0}^{\infty }\log_{2}\left(1+\gamma_{s,d}\right)f\left(\gamma_{s,d}\right)d\gamma_{s,d}\\ \; & = & \;\int_{0}^{\infty }\frac{\ln\left(1+\gamma_{s,d}\right)}{\ln2}\varpi \left[E\left(\gamma_{s,d}\right)\right]\\ \; & & \;\times \exp\left\{\frac{-{\left(\kappa_{\mathrm{LoS}}\right)}^{2}}{2{\left(\kappa_{\mathrm{NLoS}}\right)}^{2}}-\gamma_{s,d}\varpi \left[E\left(\gamma_{s,d}\right)\right]\right\}\\ \; & & \;\times I_{0}\left[2\sqrt{\frac{\varpi \left[E\left(\gamma_{s,d}\right)\right]\gamma_{s,d}}{2{\left(\kappa_{\mathrm{NLoS}}\right)}^{2}{\left(\kappa_{\mathrm{LoS}}\right)}^{-2}}}\right]d\gamma_{s,d}. \end{array}$$

By using the Gamma function $\Gamma \left(\cdot \right)$, the Bessel function $I_{0}\left[a\right]$ in (11) can be calculated as

(12) $$\begin{array}{c} I_{0}\left[a\right]=\sum_{b=0}^{\infty }\frac{{\left(\frac{a}{2}\right)}^{2b}}{b!\Gamma \left(b+1\right)}. \end{array}$$

Substituting (12) into (11), we can get

(13) $$\begin{array}{ccc} R_{s,d}\; & = & \;\frac{\varpi \left[E\left(\gamma_{s,d}\right)\right]}{\ln2}\exp\left\{\frac{-{\left(\kappa_{\mathrm{LoS}}\right)}^{2}}{2{\left(\kappa_{\mathrm{NLoS}}\right)}^{2}}\right\}\\ \; & & \;\times \sum_{b=0}^{\infty }\frac{{\left[\frac{{\left(\kappa_{\mathrm{LoS}}\right)}^{2}}{2{\left(\kappa_{\mathrm{NLoS}}\right)}^{2}}\right]}^{b}}{{\left(b!\right)}^{2}{\left\{\varpi \left[E\left(\gamma_{s,d}\right)\right]\right\}}^{-b}}\\ \; & & \;\times \int_{0}^{\infty }\frac{\ln\left(1+\gamma_{s,d}\right){\left(\gamma_{s,d}\right)}^{b}}{\exp\left\{\varpi \left[E\left(\gamma_{s,d}\right)\right]\gamma_{s,d}\right\}}d\gamma_{s,d}. \end{array}$$

On this basis, we use (14) to simplify the calculation.

(14) $$\begin{array}{c} \int_{0}^{\infty }\frac{\ln\left(1+ka\right)a^{i-1}}{\exp\left\{\beta a\right\}}da=\frac{\Gamma \left(i\right)\sum_{j=1}^{i}Y_{i-j+1}\left(\frac{\beta }{k}\right)}{\beta^{i}\exp\left\{\frac{k}{\beta }\right\}}, \end{array}$$

where $Y_{i-j+1}\left(\frac{\beta }{k}\right)$ is the improper integral [33] and can be calculated as $Y_{n}\left(x\right)=x^{n-1}\int_{x}^{\infty }\frac{\exp\left\{-t\right\}}{t}dt$, where x>0. Then, we substitute (14) into (13) and adopt (4) to derive an accurate analytical expression for $R_{\mathrm{tot}}$, as shown in (15).

(15) $$\begin{array}{ccc} R_{\mathrm{tot}}\; & = & \;\sum_{s=1}^{S}\sum_{d=1}^{D}\frac{\exp\left\{\varpi \left[E\left(\gamma_{s,d}\right)\right]-\frac{{\left(\kappa_{\mathrm{LoS}}\right)}^{2}}{2{\left(\kappa_{\mathrm{NLoS}}\right)}^{2}}\right\}}{\ln2}\\ \; & & \;\times \sum_{b=0}^{\infty }\frac{{\left[\frac{{\left(\kappa_{\mathrm{LoS}}\right)}^{2}}{2{\left(\kappa_{\mathrm{NLoS}}\right)}^{2}}\right]}^{b}}{b!}\sum_{j=1}^{b+1}Y_{b-j+2}\left\{\varpi \left[E\left(\gamma_{s,d}\right)\right]\right\}. \end{array}$$

### 3.2. Approximate Expression and Approximate Error

Equation (15) includes improper integrals and summations of infinite series, which makes direct computation difficult. Therefore, according to [17] and [33], we approximate the accurate analytical expression for $R_{\mathrm{tot}}$. Specifically, let *W* denote the truncated series. Then, given that $\sum_{b}^{\infty }\frac{K^{b}}{b!}\sum_{j=1}^{b+1}Y_{b-j+2}\left\{\frac{K+1}{E\left(\gamma_{s,d}\right)}\right\}$ has an upper ceiling approximation, we substitute it into (15). We can derive the approximate expression ${\tilde{R}}_{\mathrm{tot}}$ for $R_{\mathrm{tot}}$, which can be expressed as

(16) $$\begin{array}{ccc} {\tilde{R}}_{\mathrm{tot}}\; & = & \;\sum_{s=1}^{S}\sum_{d=1}^{D}\frac{\exp\left\{\varpi \left[E\left(\gamma_{s,d}\right)\right]-\frac{{\left(\kappa_{\mathrm{LoS}}\right)}^{2}}{2{\left(\kappa_{\mathrm{NLoS}}\right)}^{2}}\right\}}{\ln2}\\ \; & & \;\times \sum_{b=0}^{W}\frac{{\left[\frac{{\left(\kappa_{\mathrm{LoS}}\right)}^{2}}{2{\left(\kappa_{\mathrm{NLoS}}\right)}^{2}}\right]}^{b}}{b!}\sum_{j=1}^{b+1}Y_{b-j+2}\left\{\varpi \left[E\left(\gamma_{s,d}\right)\right]\right\}. \end{array}$$

Equation (16) differs from (15) in that we truncate the infinite series summation to a summation of *W* terms. In this situation, the approximate error comes from the truncation error caused by the summation of the $\left[W,\;+\infty \right)$ series, which can be expressed as

(17) $$\begin{array}{ccc} \; & & \;R_{\mathrm{tot}}-{\tilde{R}}_{\mathrm{tot}}\\ \; & = & \;\sum_{s=1}^{S}\sum_{d=1}^{D}\frac{\exp\left\{\varpi \left[E\left(\gamma_{s,d}\right)\right]-\frac{{\left(\kappa_{\mathrm{LoS}}\right)}^{2}}{2{\left(\kappa_{\mathrm{NLoS}}\right)}^{2}}\right\}}{\ln2}\\ \; & & \;\times \sum_{b=W+1}^{\infty }\frac{{\left[\frac{{\left(\kappa_{\mathrm{LoS}}\right)}^{2}}{2{\left(\kappa_{\mathrm{NLoS}}\right)}^{2}}\right]}^{b}}{b!}\sum_{j=1}^{b+1}Y_{b-j+2}\left\{\varpi \left[E\left(\gamma_{s,d}\right)\right]\right\}. \end{array}$$

For the improper integral $Y_{n}\left(x\right)=x^{n-1}\int_{x}^{\infty }\frac{\exp\left\{-t\right\}}{t}dt$, $Y_{n}\left(x\right)$ is monotonically decreasing in *n*, giving equal *x*. Therefore, the inequality (18) can be derived [34].

(18) $$\begin{array}{ccc} \; & & \;\sum_{b=W+1}^{\infty }\frac{{\left[\frac{{\left(\kappa_{\mathrm{LoS}}\right)}^{2}}{2{\left(\kappa_{\mathrm{NLoS}}\right)}^{2}}\right]}^{b}}{b!}\sum_{j=1}^{b+1}Y_{b-j+2}\left\{\varpi \left[E\left(\gamma_{s,d}\right)\right]\right\}\\ \; & < & \;\sum_{b=W+1}^{\infty }\frac{{\left[\frac{{\left(\kappa_{\mathrm{LoS}}\right)}^{2}}{2{\left(\kappa_{\mathrm{NLoS}}\right)}^{2}}\right]}^{b}}{2b!}\left(b^{2}+3b+2\right)Y_{1}\left\{\varpi \left[E\left(\gamma_{s,d}\right)\right]\right\}. \end{array}$$

It should be noted that in (18), $g\left(\gamma_{s,d}\right)$ is ignored. In addition, given $E\left(\gamma_{s,d}\right)$ and $\frac{{\left(\kappa_{\mathrm{LoS}}\right)}^{2}}{2{\left(\kappa_{\mathrm{NLoS}}\right)}^{2}}$, we have $Y_{1}\left\{\varpi \left[E\left(\gamma_{s,d}\right)\right]\right\}=D$, where *D* is a constant.

(19) $$\begin{array}{c} g\left(\gamma_{s,d}\right)=\sum_{s=1}^{S}\sum_{d=1}^{D}\frac{\exp\left\{\varpi \left[E\left(\gamma_{s,d}\right)\right]-\frac{{\left(\kappa_{\mathrm{LoS}}\right)}^{2}}{2{\left(\kappa_{\mathrm{NLoS}}\right)}^{2}}\right\}}{\ln2}. \end{array}$$

To sum up, the approximate error is influenced by *W* and has an upper bound Δ, which can be expressed as

(20) $$\begin{array}{c} \Delta \geq R_{\mathrm{tot}}-{\tilde{R}}_{\mathrm{tot}}. \end{array}$$

At present, we can only evaluate Δ through simulation experiments, as its closed-form evaluation is intractable due to the complexity of the involved random variables and integrals. This limitation will be addressed in our future work, where we plan to derive tighter analytical bounds or tractable approximations for Δ under specific system assumptions.

The proposed performance analysis scheme can be applied to smart cities [35]. Specifically, a fleet of ICVs is deployed to distribute medical supplies during a public health emergency. These ICVs must maintain continuous and reliable communication with each other and with roadside units to coordinate routes and avoid collisions. Due to the dense urban environment and high mobility, traditional communication methods suffer from spectrum congestion and unstable links. To overcome these limitations, the system applies the proposed FD-NOMA-based AAV-assisted IoV framework. AAVs act as relays to enhance signal quality, and full-duplex transmission improves spectrum efficiency. Using the proposed analytical method, engineers evaluate the total achievable rate in real-time, even under imperfect CSI. This ensures that communication among vehicles remains robust and efficient, enabling timely delivery of supplies.

Discussion: In this paper, we have made the following assumptions in order to focus on maximizing the total achievable rate:

- BER Assumption: In our analysis, we assume no bit errors during transmission, as the system operates under ideal conditions with perfect coding and decoding techniques [10]. This assumption is common in capacity analysis, especially in high SNR scenarios or with strong error correction methods such as LDPC or Turbo codes. Therefore, we do not include a detailed analysis of BER in this work, as the primary focus is on optimizing the total achievable rate.
- Delay Assumption: The delay in our model is related to the size of the data packets and determined by the transmission rate [16]. We assume that the transmission delay is inversely related to the achievable rate. In other words, maximizing the total achievable rate naturally reduces transmission delay. Since the main objective of our study is to optimize the achievable rate, delay is indirectly considered.

As mentioned above, the primary goal of this paper is to derive an accurate analytical expression for the total achievable rate over Rician fading channels, specifically for NOMA-enhanced AAV-assisted IoV systems. We also focus on the impact of imperfect channel state information (CSI) on the achievable rate. The aim is to provide a foundation for more comprehensive performance analysis, such as BER and delay, in future work.

## 4. Performance Evaluation

In this section, we use Monte Carlo simulations to demonstrate the impact of FD-NOMA on transmission performance in NOMA-enhanced AAV-assisted IoV. Specifically, the FD-NOMA technique is compared with the FD-OMA technique. Moreover, we compare the derived approximate expression with the accurate analytical expression to evaluate the performance loss due to truncation errors. Based on this, we validate the performance improvement of the derived approximate expression through running time. Furthermore, we investigate the impact of key parameters, such as the speed of ICVs and the Rician factor, on the total achievable rate. The main simulation parameters are set as follows [10,17,19,36]. The Rician factor *K* is $\left\{1,5,10,15\right\}$, the speed of ICVs is $\left[10,100\right]$ km/h, the transmission signal-to-noise ratio (SNR) is $\left[0,30\right]$ dB, and the self-interference coefficient is 0.1. In the simulation, there are 10 ICVs (i.e., V=10) traveling on the highway. The next-generation nodes B (gNBs) are deployed at intervals of 1.5 km along the highway, and they are 200 m away from the highway. The antenna uses a directional sector beam (with a horizontal coverage of 60–120°) to cover long-distance roads. The communication frequency is 5.9 GHz. The antenna gains of the AAV and the ICV are 18 dBi and 5 dBi, respectively, which are utilized to compensate for path loss and improve resistance to Doppler shift. The power allocation follows an arithmetic descending order distribution. In the Monte Carlo experiment, we use a 12-core, 16-thread 2.1 GHz processor, 32 GB of memory, and a 64-bit Windows 10 operating system.

**Figure 2:**
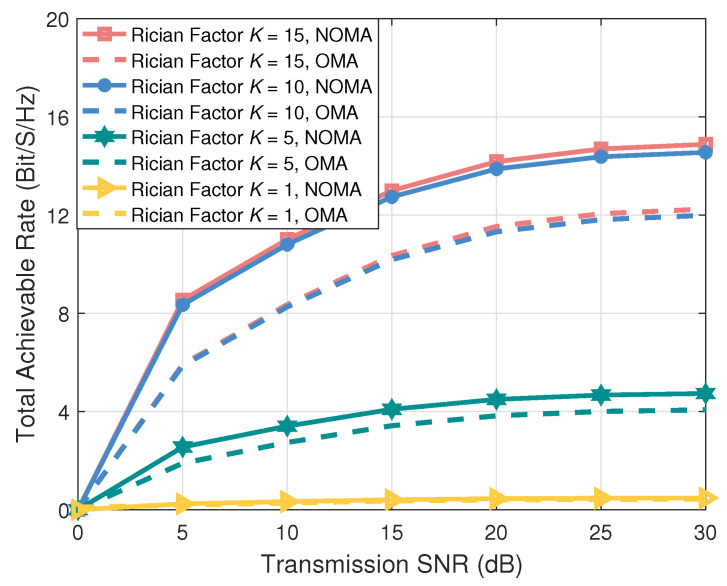
The total achievable rate versus the transmission SNR.

As shown in Figure 2, by varying the transmission SNR, we compare the performance difference in the total achievable rate between FD-NOMA and FD-OMA. Simulation results demonstrate that FD-NOMA offers higher total achievable rate compared to FD-OMA. This is due to the use of superposition coding and SIC techniques in FD-NOMA, which allow multiple ICVs to share the same time-frequency resources, thereby improving the spectrum efficiency of V2X communication. Therefore, in AAV-assisted IoV, FD-NOMA can simultaneously meet the demands for low latency and high reliability. Additionally, as the Rician factor increases, the simulation results show that the total achievable rate of both FD-NOMA and FD-OMA increases. The reason is that an increase in the Rician factor indicates a stronger LoS component, which can improve the signal quality. Furthermore, we conduct a comparative analysis using Rician factors of 15 and 10. Although the Rician factor decreased from 15 to 10, the total achievable rate of FD-NOMA remains higher than that of FD-OMA. This indicates that even in more unfavorable channel conditions, FD-NOMA continues to outperform FD-OMA, thereby confirming the necessity of employing FD-NOMA techniques in the AAV-assisted IoV.

**Figure 3:**
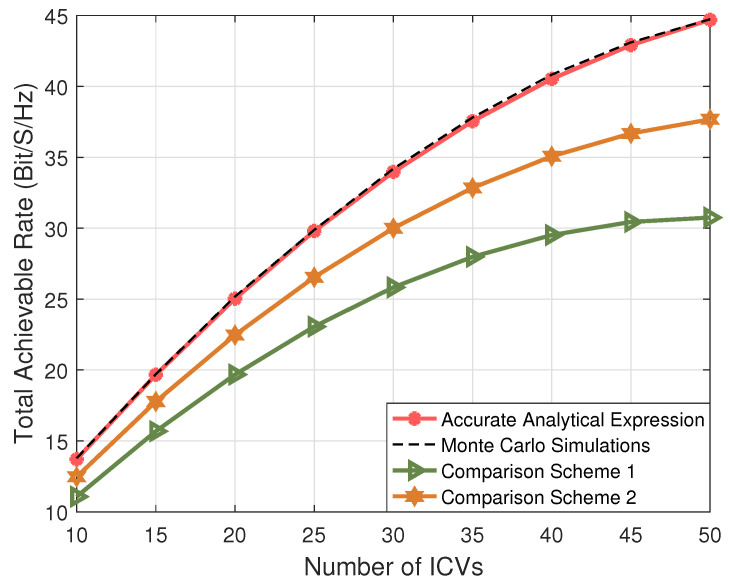
The total achievable rate versus the number of ICVs.

Figure 3 shows the total achievable rate versus the number of ICVs. In our simulations, we compare the NOMA-enhanced AAV-assisted vehicular communication scheme with two existing studies: Scheme 1 [25] and Scheme 2 [19]. We can find that the NOMA-enhanced AAV-assisted vehicular communication scheme proposed in this paper achieves a higher total achievable rate compared to existing schemes 1 and 2. This is because, although Scheme 1 utilizes UAVs, it still employs the traditional orthogonal multiple access scheme, which limits its performance. Furthermore, Scheme 2 applies the NOMA technique but fails to fully exploit the mobility of UAVs, thus not maximizing the potential gains from the UAV’s dynamic positioning. Additionally, comparing the derived accurate analytical expression with Monte Carlo results shows a good match, which validates the correctness of the theoretical analysis.

**Figure 4:**
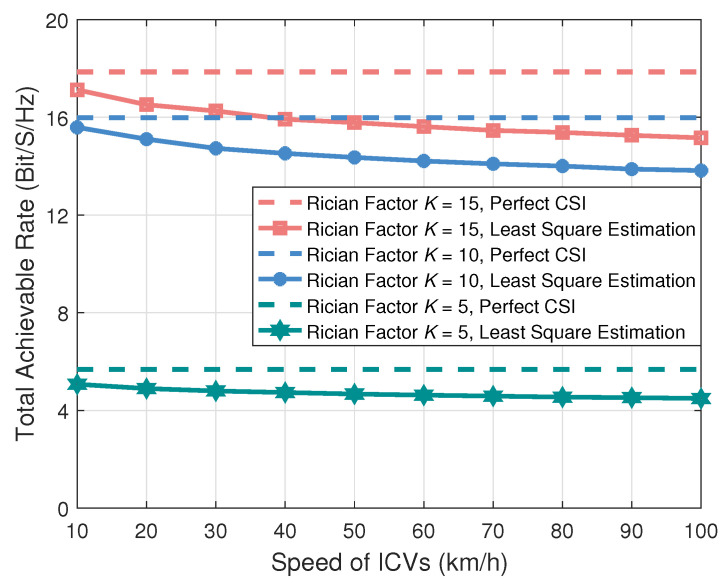
The total achievable rate versus the speed of ICVs.

Figure 4 plots the impact of the speed of ICVs on the total achievable rate. It can be observed that as the speed of ICVs increases, the total achievable rate of AAV-assisted IoV exhibits a downward trend. This is because we use the LS method to estimate CSI. However, the high-speed movement of ICVs causes rapid channel variations, which exacerbate CSI estimation errors. The LS estimation method is sensitive to noise and interference, and in high-speed scenarios, it struggles to accurately track air-to-ground channel variations, leading to reduced CSI estimation accuracy. In such situations, inaccurate CSI estimation further affects the implementation of SIC techniques, thereby reducing the total achievable rate. In the future, deep learning methods could be employed to train models using historical channel data, enabling intelligent prediction and estimation of time-varying channels, thus mitigating the impact of imperfect CSI on total achievable rate.

**Figure 5:**
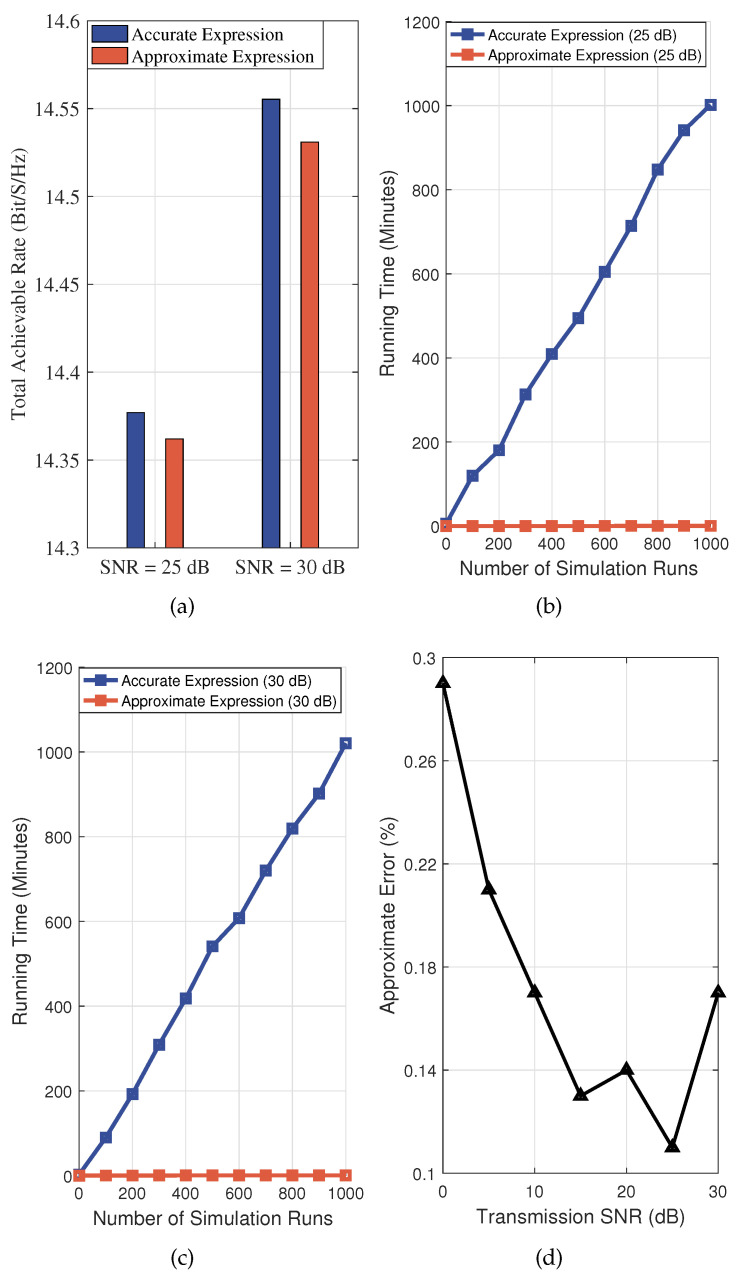
The accurate analytical expression versus the approximate expression. (**a**) Total achievable rate comparison. (**b**) Running time $\left(\mathrm{SNR}=25\;\mathrm{dB}\right)$. (**c**) Running time $\left(\mathrm{SNR}=30\;\mathrm{dB}\right)$. (**d**) Approximate error versus the transmission SNR.

Figure 5a depicts the performance difference in total achievable rate between the accurate analytical expression and the approximate expression. We can find that, although the approximate expression simplifies the computation by truncating the infinite series, the difference between its results and those of the accurate analytical expression is negligible, with the maximum approximate error not exceeding 0.5%. Then, Figure 5b,c further compare the performance of the two expressions in terms of running time. Under the same simulation environment, the running time of the accurate analytical expression is significantly higher than that of the approximate expression, with the former requiring approximately 1000 times more computation time than the latter. These results validate the effectiveness of the derived approximate expression, demonstrating that it significantly reduces computational complexity while maintaining computational accuracy. In NOMA-enhanced AAV-assisted IoV, the use of the approximate expression can effectively balance computational accuracy and operational efficiency, thus better satisfying the practical requirements of vehicular applications. In addition, Figure 5d depicts the approximate error versus the transmission SNR. It can be observed that, regardless of variations in the transmission SNR, the approximation error consistently remains below 0.5%. This demonstrates the accuracy and robustness of the proposed approximation method.

## 5. Conclusions

The integration of AAVs and FD-NOMA was investigated in this letter, aiming to improve the achievable rate of V2X communications in IoV. Specifically, in NOMA-enhanced AAV-assisted IoV, we proposed an accurate analytical expression of the total achievable rate over Rician fading channels. Then, to simplify the calculation, an approximate expression was derived, based on which a closed-form solution for the approximation error was obtained. Our analysis shows that the approximation error is determined by the truncated series. Finally, the simulation results show that the proposed accurate analytical expression matched well with the derived approximate expression, and the maximum approximate error was less than 0.5%. Meanwhile, the running time was greatly reduced by the derived approximate expression. In addition, the simulation results also demonstrated that FD-NOMA improved the total achievable rate compared to FD-OMA. In the future, we will further analyze the impact of AAV trajectories and the movement of ICVs on the total achievable rate.

## Figures and Tables

**Table 1:** Notations used in this paper.

Parameter	Definition
*U*	Number of autonomous aerial vehicles (AAVs)
*V*	Number of intelligent connected vehicles (ICVs)
*S*	Number of source nodes (SNs)
*D*	Number of destination nodes (DNs)
$H_{\mathrm{down}}$	Downlink channel matrix between *S* SNs and *D* DNs
$h_{d}^{\mathrm{down}}$	Channel matrix from *S* SNs to the *d*-th SN
$h_{s,d}^{\mathrm{down}}$	Downlink channel fading coefficient from the *s*-th SN to the *d*-th DN
$z_{\mathrm{down}}$	Downlink received signal
$P_{\mathrm{down}}$	Downlink power matrix
$x_{\mathrm{down}}$	Downlink transmission signal matrix
$n_{\mathrm{down}}$	Downlink noise matrix
$\sigma_{\mathrm{down}}^{2}$	Downlink noise power
$I_{D}$	Identity matrix
$x_{\mathrm{up}}$	Uplink transmission signal matrix
$n_{\mathrm{up}}$	Uplink noise matrix
$\sigma_{\mathrm{up}}^{2}$	Uplink noise power
$R_{\mathrm{tot}}$	Total achievable rate

## Data Availability

Data are contained within the article.
